# Isolated Cystic Duct Injury Caused by a Gunshot Wound: A Case Report of an Extremely Rare Finding

**DOI:** 10.7759/cureus.98592

**Published:** 2025-12-06

**Authors:** Andrés David De León Murillo, Pedro Antonio Plaza Ricardo, Indira Tatiana Contreras Vanegas, Miguel Angel Fuentes Iglesias, Sergio A Tavera, Tatiana Paola Pérez García, Alejandro Alfonso Bedoya Rinaldi

**Affiliations:** 1 General Surgery, Universidad Libre, Barranquilla, COL; 2 General Surgery, Clínica Keralty, Ibagué, COL; 3 Medicine, Universidad Metropolitana, Barranquilla, COL; 4 Surgery, Universidad Metropolitana, Barranquilla, COL

**Keywords:** abdominal trauma, emergency, extrahepatic biliary trauma, extrahepatic biliary tree trauma, trauma

## Abstract

Extrahepatic biliary tree injuries are uncommon, but isolated extrahepatic biliary tree injuries are extremely rare and are usually associated with other injuries. The most common associated injuries include liver, spleen, and/or duodenal injuries. Due to their rarity and generally insidious onset of symptoms, extrahepatic biliary tree injuries are often overlooked during the initial trauma evaluation, which can lead to significant associated morbidity. Their management, considering hemodynamic stability and imaging findings, includes nonoperative and surgical management, which may include minimally invasive (laparoscopy), open surgery, and/or endoscopy. We present the case of a 28-year-old male patient admitted with a gunshot wound to the abdomen. Surgical findings included a laceration of the cystic duct, with no associated injuries. A cholecystectomy is performed. Following surgery, the patient made satisfactory postoperative progress and was subsequently discharged.

## Introduction

Extrahepatic biliary tree injuries are uncommon, occurring in 0.1% of adult trauma admissions, 0.009% of pediatric trauma admissions [1], and 2.8-7.4% of patients with blunt hepatic trauma [2]. Isolated injuries to the extrahepatic biliary tree are extremely rare, occurring in only 2-3% of cases [1], and are usually associated with injuries. The most common associated injuries include liver injury (91% of cases) and splenic and duodenal injuries (both in up to 54% of cases) [3]. Blunt trauma is the most common cause of extrahepatic biliary tree injuries, except for the gallbladder, which is most frequently injured by penetrating trauma, in up to 89% of cases [1,4]. This low incidence may be secondary to the mechanical protection provided by the liver [3,4].

Due to their rarity and generally insidious onset of symptoms, extrahepatic biliary injuries are often overlooked during the initial trauma evaluation, which can lead to significant associated morbidity [5,6]. Biliary injury may be evident at the time of initial surgery with the presence of bile drainage through the hepatic parenchyma, the extrahepatic biliary tree, or the pancreas. Postoperatively, biliary injury may be evidenced by the presence of a biliary fistula, biliary peritonitis, bilioma, or biliary stricture. Rarely, a biliary injury may present as a biliopleural, bronchopleural, or biliovenous fistula, or hemobilia [7].

Extrahepatic biliary tract injuries are categorized according to the classification proposed by the American Association for the Surgery of Trauma (AAST), which ranges from Grade I to Grade V, corresponding to contusion or hematoma of the gallbladder and/or portal triad to transection of more than 50% of the common hepatic duct and/or common bile duct, combined injury to the right and left hepatic ducts, and/or injury to the intrapancreatic or intraduodenal bile duct. On the other hand, the World Society of Emergency Surgery (WSES) proposes a classification that categorizes these injuries into mild (WSES class I), moderate (WSES class II), and severe (WSES classes III and IV) [1].

Due to the high percentage of associated injuries, patients with extrahepatic biliary tree injuries present with peritonitis and shock. Hemodynamic instability occurs in up to 10-44% of patients, and in these patients, surgery is the imperative indication [1]. The management of these injuries will depend on the presence or absence of associated injuries and their degree; this can range from nonoperative management to endoscopic or surgical management [1]. Damage control surgery is reported in 20-63% of cases, particularly in patients with associated vascular injuries and/or high-grade pancreaticoduodenal injuries [1]. Gallbladder injuries are almost always managed with cholecystectomy [5].

Given the advent of nonoperative management of patients with abdominal trauma in general and the insidious presentation of these injuries, it is important to always consider them in the patient's evaluation [4].

## Case presentation

We present the case of a 28-year-old male patient who was admitted to the emergency department 30 minutes after sustaining a gunshot wound to the abdomen during a fight. Upon arrival, the patient reported severe abdominal pain, accompanied by abdominal distension and signs of peritoneal irritation, symptoms present from the outset.

On admission, the patient was hemodynamically stable, with a blood pressure of 110/70 mmHg, a heart rate of 95 bpm, a respiratory rate of 18 breaths per minute, and an oxygen saturation of 98% on room air. Physical examination revealed an entry wound in the epigastrium, with no exit wound.

Following the primary and secondary assessments established by the current ATLS (Advanced Trauma Life Support) protocol, initial laboratory tests were performed, including a complete blood count and red blood cell reserve determination. Due to the presence of peritoneal irritation and the suspicion of intra-abdominal injury, an emergency exploratory laparotomy was indicated.

**Figure 1:**
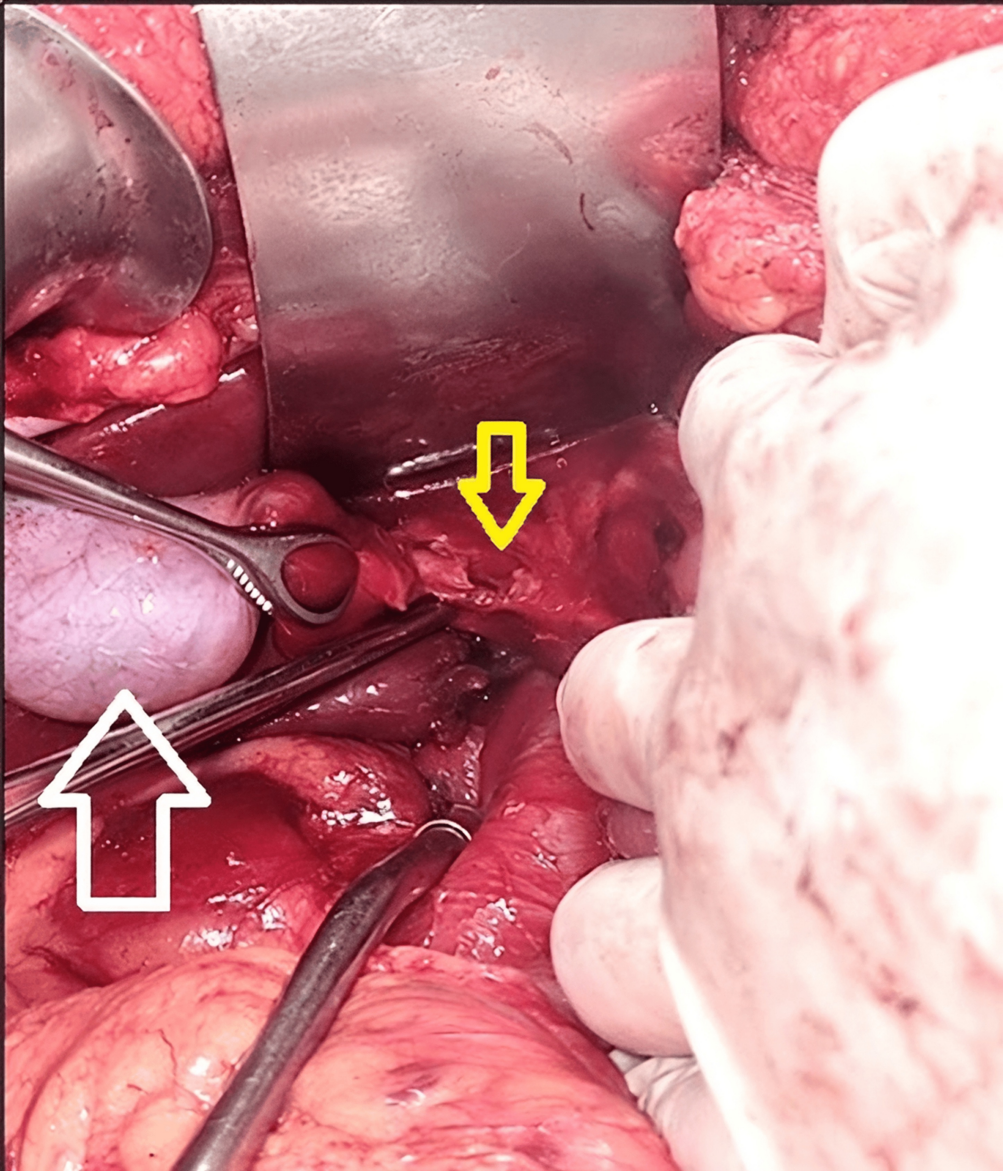
Exposure of the gallbladder and cystic duct. Yellow arrow: cystic duct with laceration greater than 50%. White arrow: gallbladder.

**Figure 2:**
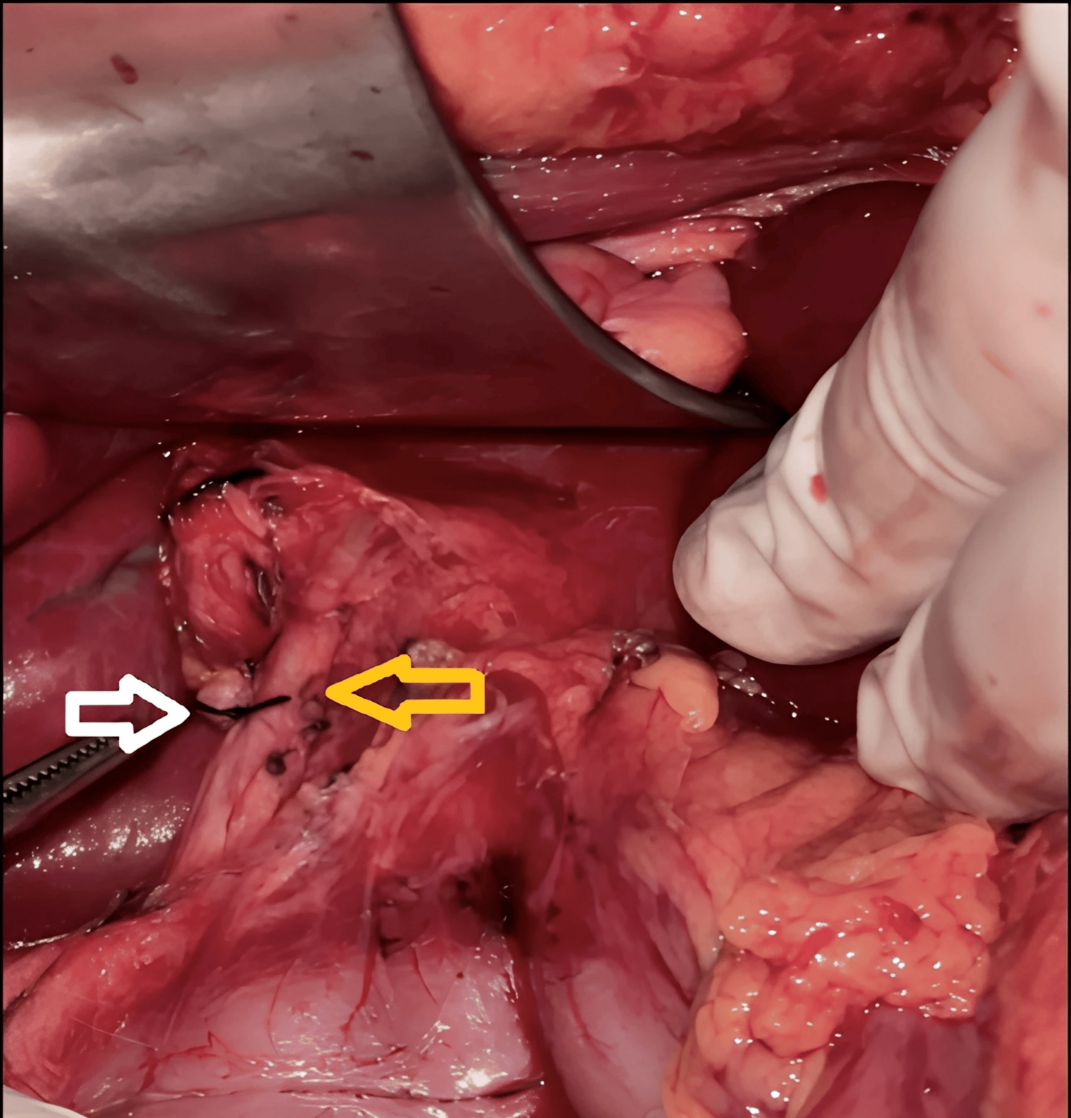
Cholecystectomy performed. White arrow: ligated stump of the cystic duct. Yellow arrow: common bile duct.

In the operating room, approximately 150 ml of bilioperitoneum and more than 50% transection of the cystic duct were found (Figure 1), with no evidence of associated injuries. After obtaining the critical Strassberg safety projection, a total cholecystectomy was performed (Figure 2).

The patient was discharged in stable condition and was evaluated in the outpatient clinic 10 days later, without complications. Further follow-up was conducted until day 30 post-procedure, with no mortality or adverse events recorded during this period.

## Discussion

Traumatic injuries of the biliary tree are rare, with an estimated incidence of less than 0.1% of trauma-related hospital admissions. This low frequency is due to its anatomical location, with iatrogenic injuries and those associated with severe blunt hepatic trauma being more common [7]. Isolated injuries of the extrahepatic biliary tree secondary to penetrating trauma represent a minority of reported cases, and their preoperative diagnosis is often challenging due to the absence of specific clinical manifestations and the frequent association with other visceral injuries [3].

The classification of extrahepatic biliary tree injuries, such as that proposed by the AAST or the WSES, helps guide management [1]. The AAST scale proposes five grades that stratify the type and severity of biliary injuries from least to most severe. This stratification is fundamental for surgical decision-making. In Grade I, injuries correspond to contusions or hematomas of the gallbladder or contusions of the portal triad. Grade II may involve partial avulsion of the gallbladder from the hepatic bed or lacerations/perforations of the gallbladder itself. Grade III injuries include complete avulsion of the gallbladder from the hepatic bed or laceration of the cystic duct. More complex injuries, such as Grades IV and V, involve partial or complete lacerations of the right or left hepatic ducts or the common bile duct, including combined or intrahepatic injuries [1]. Additionally, the WSES classification provides a slightly more comprehensive framework, categorizing injuries into four classes while considering the AAST grading and the patient's hemodynamic status [1].

From a surgical perspective, in cases of gallbladder injury, cholecystectomy remains the standard treatment [5]. In this clinical case, the location of the injury at the cystic duct justified this intervention as the most direct option to prevent biliary complications, such as fistulas, bilomas, or biliary peritonitis [7].

Immediate intraoperative diagnosis is crucial, as unidentified injuries may evolve into complications such as biliary fistulas, bilomas, or biliary peritonitis, which significantly increase morbidity and mortality [4].

Gallbladder injuries in the context of trauma are very uncommon, and when they occur, they happen alongside concomitant injuries. Isolated injuries of the cystic duct specifically are extremely rare and have been described in only a few case reports, in which no epidemiological data are available. Jain et al. reported an isolated cystic duct injury concomitant with a diaphragmatic and intestinal injury [8]. On the other hand, Cho and Lim reported a transection of the cystic duct along with the cystic artery in the context of blunt trauma [9].

This case highlights the importance of considering cystic duct injury as a differential diagnosis in patients with abdominal trauma undergoing surgical intervention, either minimally invasive (laparoscopy) or open (exploratory laparotomy), as in this case, where biliperitoneum was found during the procedure. Similarly, the usefulness of extended focused assessment with sonography in trauma (E-FAST) is emphasized as an initial, rapid, repeatable, and effective tool for urgent surgical decision-making in abdominal trauma patients, particularly in institutions where this resource is available [1,5].

Finally, this report contributes to the existing literature by documenting an isolated cystic duct injury secondary to penetrating trauma in a young patient without the involvement of adjacent organs, a situation reported in less than 3% of cases in recent series [3]. Its clinical presentation, diagnostic approach, and surgical management may serve as a reference in similar scenarios, especially in centers where decision-making time and resources are limited.

## Conclusions

Despite the infrequency of isolated injuries to the extrahepatic biliary tree, they should not be ruled out in patients admitted to the emergency department in the context of trauma and whose intraoperative finding is biliperitoneum. Furthermore, considering that intra- and/or extrahepatic biliary tract injuries are often associated with other concomitant injuries, such as hepatic, duodenal, and splenic injuries, the latter being the most common, verifying the adequate integrity of these structures should be practically mandatory.
